# Supplementary material for: Soft jamming of viral particles in nanopores
Source: Nat Commun. 2024 Jul 23;15:6180. doi: 10.1038/s41467-024-50059-9 (PMC11263580; doi:10.1038/s41467-024-50059-9)
Supplement: Supplementary file 1 — Supplementary Information [file 41467_2024_50059_MOESM1_ESM.pdf]

Supplementary Information:

Soft jamming of viral particles in nanopores

Léa Chazot-Franguiadakis[1], Joelle Eid[2], Gwendoline Delecourt[3], Pauline J. Kolbeck[1,4,5], Saskia Brugère[1], Bastien Molcrette[1,6], Marius Socol[2], Marylène Mougel[2], Anna Salvetti[7], Vincent Démery[1,8], Jean Christophe Lacroix[9], Véronique Bennevault[3,10], Philippe Guégan[3], Martin Castelnovo[1] and Fabien Montel[1]*

[1]*Laboratoire de Physique, UMR CNRS 5672, ENS de Lyon, Université de Lyon, Lyon, France.*

[2] *Institut de Recherche en Infectiologie de Montpellier, UMR CNRS 9004, Université de Montpellier, Montpellier, France.*

[3] *Institut Parisien de Chimie Moléculaire, UMR CNRS 8232, Sorbonne Université, Paris, France.*

[4] *Department of Physics and Center for NanoScience, LMU Munich, 80799 Munich, Germany.*

[5] *Department of Physics and Debye Institute for Nanomaterials Science, Utrecht University, 3584 CC Utrecht, The Netherlands.*

[6]*Department of Functional Genomics and Cancer, Institute of Genetics and Molecular and Cellular Biology, UMR CNRS 7104, University of Strasbourg, Illkirch, France.*

[7] *Centre International de Recherche en Infectiologie, UMR CNRS 5308, Université de Lyon, INSERM, Lyon, France.*

[8] *Gulliver, UMR CNRS 7083, ESPCI Paris, Université PSL, Paris, France.*

[9] *Université Paris Cité, ITODYS, CNRS, F-75006 Paris, France.*

[10] *University of Evry, Evry 91000, France.*

E-mail: fabien.montel@ens-lyon.fr

Supplementary Methods

Viral Particle Production

Human Immunodeficiency Virus (HIV-1) and Murine Leukemia Virus (MLV) were obtained from Marylène Mougel lab in IRIM (Montpellier, France). HIV were obtained in the form of virus-like particle from monoclonal human HeLa cells stably expressing Gag-GFP (more information on HIV production is available in Ref[1,2]). More precisely, Green Fluorescent Protein (GFP, $\lambda_{exc} = 488nm/\lambda_{em} = 507nm$) was fused in C-terminal of Gag protein[3] . MLV were obtained both in the form of VLP and complete viruses from NIH3T3 mouse embryonic fibroblast cells, expressing independently Gag-GFP[3] and MLV-GFP (unpublished).

Hepatitis B virus (HBV) and Adeno-associated virus (AAV) were obtained from Anna Salvetti lab in CIRI (Lyon, France). HBV capsids were purified from a viral stock of infectious HBV particles (genotype D) concentrated on a 20% sucrose cushion. The removal of HBV envelops made the particles non-infectious. As for AAV, we used two serotypes AAV-8 and AAV-9, that were generated by calcium phosphate transfection of HEK-293 cells[4] . HBV and AAV were fluorescently labelled using YOYO-1 (Molecular Probes, 1 mM in DMSO, $\lambda_{exc} = 491nm$ $/\lambda_{em} = 509nm$). Incubation time is about 10 min at room temperature.

For all those viral particles, detailed protocols can be found in Supporting Information of a previous article[5] .

Dilute solutions of viral particles in Tris-EDTA buffer (10mM if Tris-KCl, 1 mM of EDTA, pH=7.5) were prepared. Serum Fetal Bovin (FSB) can be added to 10% (if no serum was present in the initial solution). Final solutions (in the range of 10^6 particles/mL, $50\mu L$) were filled in the cis chamber. All concentrations were determined using a previously published concentration quantification method with our ZMW system[5] .

Fluorescent labelling

The labelling process varied depending on the target of the fluorescent label. Two labelling strategies have been employed: targeting of genetic material (DNA, RNA) using YOYO-1 or fluorescent labelling inherent to virus production (e.g Gag-GFP), as illustrated in Supplementary Figure 1.A of the article. The two markers were chosen to have excitation and emission wavelengths compatible with the experimental setup. Moreover, the fluorescent markers used did not modify the properties of the viral particles. They were passive markers, with no interaction with the nanopores, and did not alter the translocation of the viral particles.

-YOYO-1. More precisely, YOYO-1 is used to fluorescently labelled genetic material (DNA or RNA). It is a green intercalant belonging to the cyanine family. Alone in solution, YOYO-1 has a low quantum yield and can be considered as almost non-fluorescent, whereas when bound to DNA/RNA with which it forms a complex, its yield increases 1000-fold.[6–8] Its fluorescence spectrum, indicates excitation and emission wavelengths of 491 nm and 509 nm respectively (Supplementary Figure 1.B). YOYO-1, whose chemical structure is shown in Supplementary Figure 1.A, binds to DNA by bis-intercalation of its two chromophore units.

YOYO-1 is purchased in 200 μL solution at 1 mM in DMSO from Molecular probes. We optimized the YOYO-1 to DNA ratio to ensure DNA chain saturation (1 intercalant per 4 DNA bases) without increasing background fluorescence. Moreover, the addition of YOYO-1 leads to an approximately 38% increase in DNA contour length when the chain is saturated with YOYO-1. This corresponds to a new length per base of around 0.47 nm when YOYO-1 is saturated, compared with 0.34 nm without YOYO-1.[7,9,10] Nevertheless, the use of YOYO-1 does not alter the persistence length of DNA. Furthermore, the value of the equilibrium dissociation constant between DNA and YOYO-1 ranges from 700 pM to 12 nM, equivalent to a free enthalpy of reaction of around 18-21 k_BT.[11–13]

We have evidenced that YOYO-1 is able to label DNA inside AAV and HBV viruses,

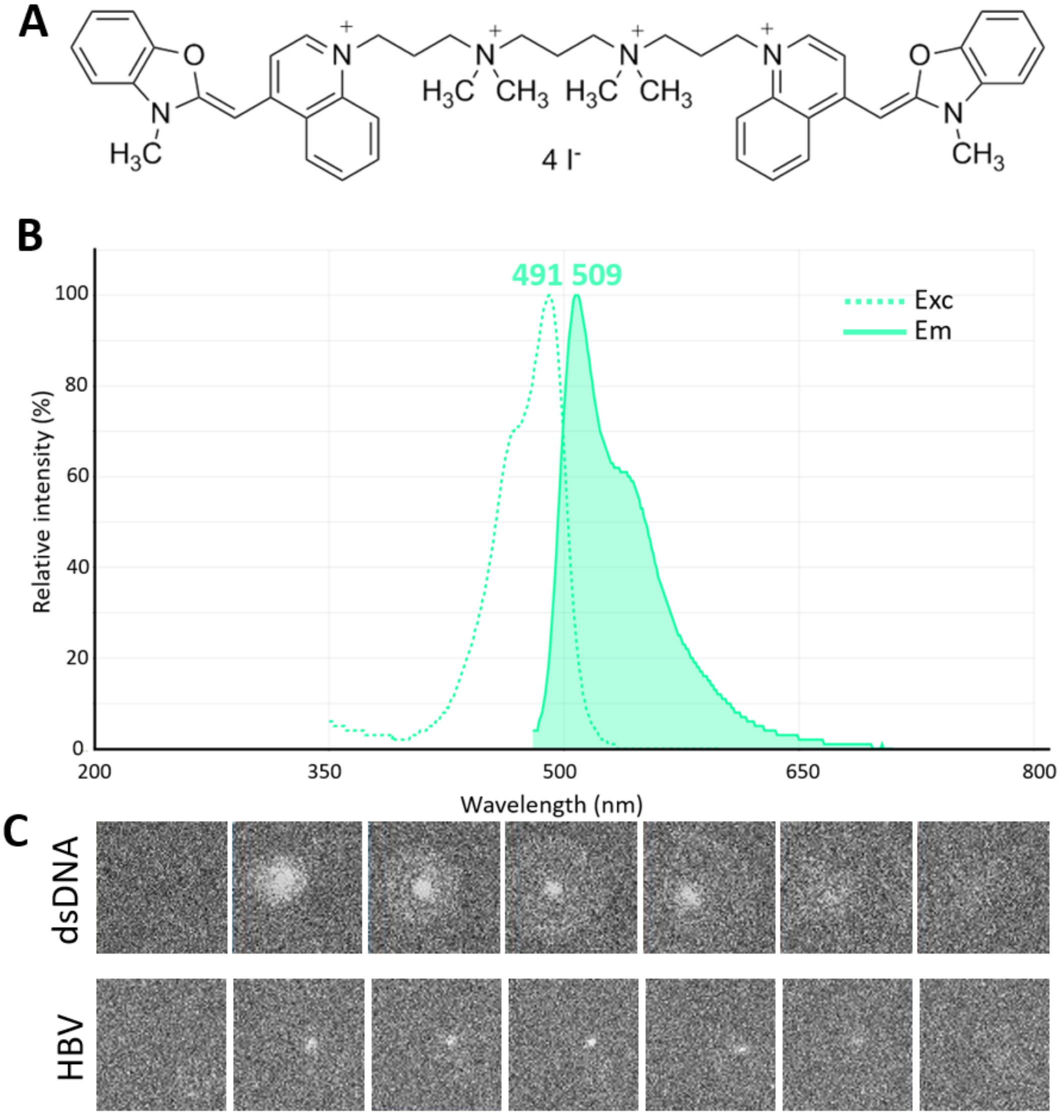

Supplementary Figure 1: **A. Chemical formula of YOYO-1. B. Emission and excitation spectra of YOYO-1.** Produced with SpectraViewer. **C. Images corresponding to translocation exit event of dsDNA and HBV labelled using YOYO-1.** (up) dsDNA corresponded to -DNA (linear double stranded DNA of 48 502 pb). (down) HBV capsids contained double stranded DNA partially circular of 3200 pb. Pores 200 nm. Image size: 15 µm. Frame rate: 33 fps.

by effectively penetrating their viral capsids.[5] One possible mechanism for this penetration is through the capsid respiration process. Importantly, the labelling occurring inside the capsid does not affect the interaction of the virus with the nanopore. Furthermore, we also conducted a comparative analysis of images from free DNA in solution versus DNA encapsulated within viral capsids (Supplementary Figure 1.C). The spatial distribution of fluorescence signals allows us to distinguish between these two conditions.

-GFP. Regarding inherent fluorescent labelling, we relied on Gag-GFP for both HIV (VLP) and MLV particles. More precisely, for HIV (VLP) and MLV, fluorescent labelling is achieved using GFP fused to the C-terminal position of the Gag structural protein. In the case of the full-length MLV virus, GFP is incorporated into the center of the Gag polyprotein. The fluorescence spectrum of GFP is shown in Supplementary Figure 2.B, with excitation and emission wavelengths of 488 nm and 507 nm respectively. The natural GFP protein has 238 amino acids and a molecular mass of 27 kDa (Supplementary Figure 2.A). Despite this high molar mass, the addition of GFP to Gag protein does not alter its assembly and release properties.[14,15]

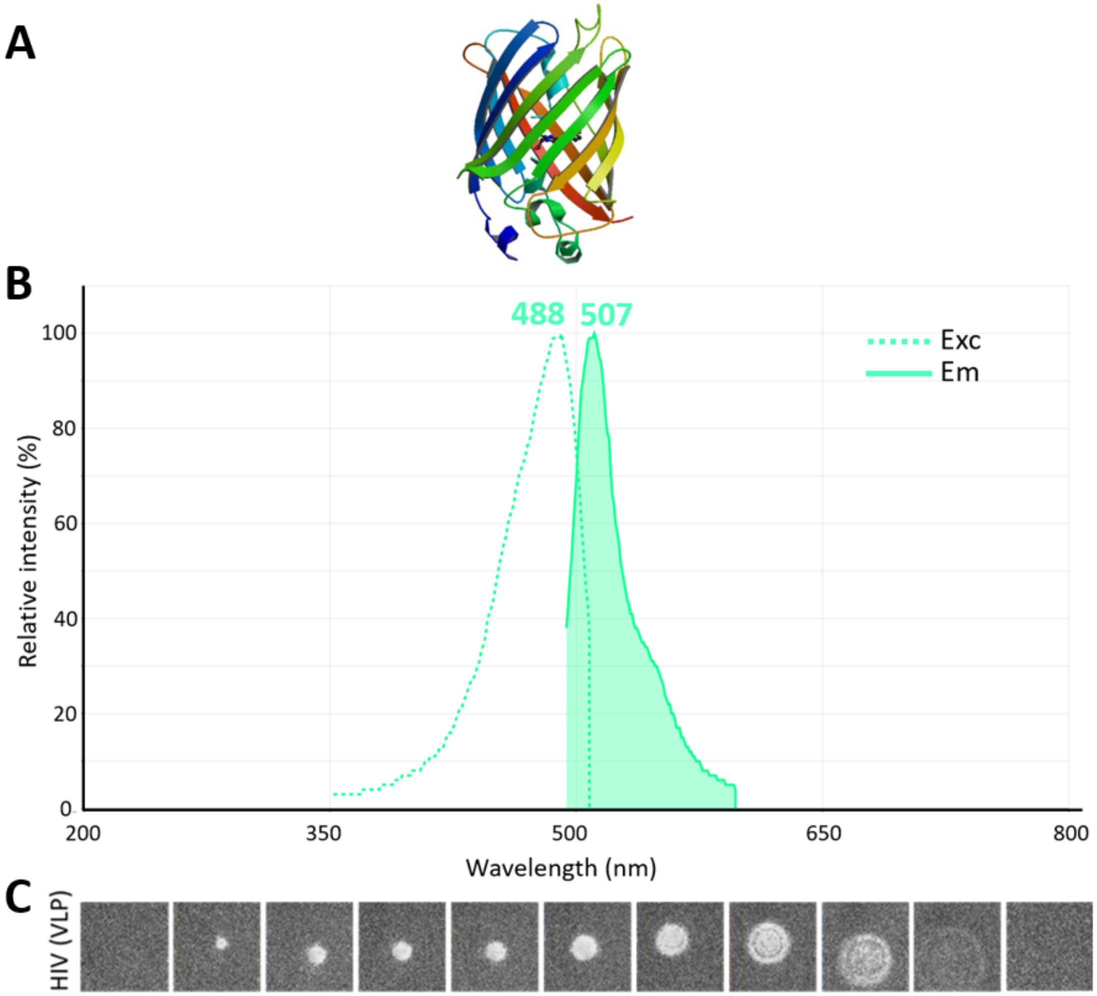

Supplementary Figure 2: **A. Structure of the natural GFP protein from the jellyfish Aequorea victoria**, taken from Ref.[16] **B. Emission and excitation spectra of GFP, produced using with SpectraViewer. C. Images corresponding to translocation exit event of HIV (VLP) labelled using Gag-GFP.** Pores 400 nm. Image size: 15 µm. Frame rate: 33 fps.

Fluorescent Nanobeads

Two types of fluorescent polystyrene nanobeads were used in this study:

- Fluorescent carboxyl polystyrene nanobeads ($SPHERO^{TM}$ UV/Yellow, *Spherotech Inc*). Two samples were used: small nanobeads of radius $R_{beads} = 38 \pm 11nm$ (size was measured by DLS equipment (DLS Zetasizer Nano-S, Malvern InstrumentTM)) and initial concentration of 8.10^{13} particles/mL; and larger nanobeads of radius $R_{beads} = 130 \pm 35nm$ (size was provided by the supplier) and initial concentration of 1.10^{12} particles/mL.Because of the presence of the carboxyl groups, theses nanobeads have limited interaction with the pore surface (polycarbonate and gold).

- Fluorescent amino polystyrene nanobeads ($SPHERO^{TM}$ Yellow, *Spherotech Inc*, 4.10^{15} particles/mL). Radius was provided by the supplier, $R_{beads} = 85 \pm 25nm$. The presence of the amino groups, induced attractive interaction of these nanobeads with the pore surface.

Dilute solutions of nanobeads in Tris-EDTA buffer (10mM if Tris-KCl, 1 mM of EDTA, pH=7.5) were prepared. Serum Fetal Bovin (FSB) was added up to 10%.

Nanoporous Membranes

The nanoporous membranes were commercially available track-etched membranes (Whatman, GE Polycarbonate, delivered by VWR) of four different pore diameters: 80 nm (reference WHAT10419306); 100 nm (reference WHAT10419506); 200 nm (reference WHAT10417006) and 400 nm (reference WHAT10417106). They correspond to dense arrays of cylindrical nanopores produced through heavy ion irradiation. Density, orientation and diameter of the nanopores are controlled. For this study, we used membranes of different diameters and pore densities (Supplementary Table 1). They were coated with a thin layer of gold (EVA 300 Alliance Concept evaporator, thickness 50 nm, speed of deposit 0.1 nm/s) in order to enable optical detection of translocation events. Effective diameters of the pores were checked by

Scanning Electron Microscopy (values * were determined in a previous article[9] , and other using SEM of Laboratoire de Physique, ENS Lyon, France).

Supplementary Table 1: **Specifications of track-etched membranes used in this study** (source Whatman, *GE Healthcare*) and * Ref[9] .

Nominal diameter of the pore (nm) (+0%,-20%)	80	100	200	400
Effective diameter of the pore (nm)	88±0.7*	90±0.7*	220±2*	395±15
Nominal thickness of the membrane (μm) (±10%)	6	6	10	10

Membrane Modifications

Membrane grafted with poly(2-methyl-2-oxazoline)s, PMeOx

Polymer synthesis Polymers were synthesized at the Institut Parisien de Chimie Moléculaire (IPCM) with Philippe Guégan and Véronique Bennevault. The polymerization of 2-methyl-2-oxazoline is well controlled (through process of Cationic Ring Opening Polymerization, CROP) and allows to introduce an aminophenyl end on the polymers via the terminating agent. This extremity will be used for the grafting of polymers on nanoporous gold membranes. The terminating agent, used to introduce an aminophenyl group at the end of the chain, was 1-(4-aminophenyl)piperazine and the initiator was allyl bromide. The polymerizations were carried out at 80°C, in acetonitrile (ACN). The reagents (monomers, initiator, terminating agent) had been preliminary cryo-distilled in order to be introduced in the reaction medium without any trace of water and under inert atmosphere. After the synthesis, polymers were dialyzed against methanol, using a regenerated cellulose membrane (Repligen, Spectra/Pore 6, Standard Regenerated Cellulose) with a cutoff at 1000 Da, in order to remove excess of terminating agent. The obtained polymers were characterized using ^1H NMR (Bruker Nanobay 300 MHz, in $CDCl_3$ solvant) and Size Exclusion Chromotagraphy (SEC, in N,N-dimethylformamide, with poly(methyl methacrylate) standards). For this high molar mass polymers, the degree of polymerization (Xn) could not be determined by NMR and

the SEC value was underestimated due to polymer adsorption to SEC column and PMMA calibration issues[17] . Experimental conditions and characterizations are detailed in Tab 2, and more information can be found in Ref[18,19] .

Supplementary Table 2: **Overview of PMeOx synthesized and its characteristics.** $[M]_0/[A]_0/[Q]$, monomer/initiator/terminating agent ratios. ^{A} Conversion rate determined by ^1H 300 MHz NMR in $CDCl_3$, reaction medium. ^{B} Theoretical degree of polymerization, $Xn = [M]_0 \times$ Conversion$/[A]_0$. ^{C} Xn determined by SEC (DMF, 60°C, standard PMMA), dialyzed polymer. ^{D}Dispersity.

$[M]_0/[A]_0/[Q]$	Time (days)	p^A(%)	Xn th^B	Xn CES^C	Đ^D
530/1/5	11	99	526	387	1.7

Electrografting of PMeOx on gold nanoporous membranes. The grafting of the polymers (PMeOx) on the gold nanoporous membranes was carried out by electrochemical reduction at the laboratory of Interfaces Traitements Organisation et Dynamique des Systèmes (ITODYS) with Jean-Christophe Lacroix. Grafting was based on the generation of a diazonium salt *in situ* from the aminophenyl end of the polymers, in the presence of an acid ($HClO_4$) and a nitrite compound ($NaNO_2$). The reduction of the diazonium salt enabled the formation of a covalent bond between the polymer and the gold surface[19] , as represented in Supplementary Figure 3. Grafting was characterized with our experimental setup, by using the translocation of double-stranded DNA and the suction model.[9,18,20] Relying on the suction model, the thickness of the grafted layer was measured : 8.9 ± 1 nm. The thickness of the grafted layer is therefore superior to the Debye length (around 3 nm in our experimental buffer conditions).

Passivation of nanoporous membranes

The nanoporous membranes were passivated using pluronic (F127) which is a triblock copolymers of polyethylene oxide (PEO) and polypropylene oxide (PPO) ($PEO_{100} - PPO_{65} - PEO_{100}$) and a non-ionic surfactant, commercially available (*Sigma-Aldrich*). Pluronic

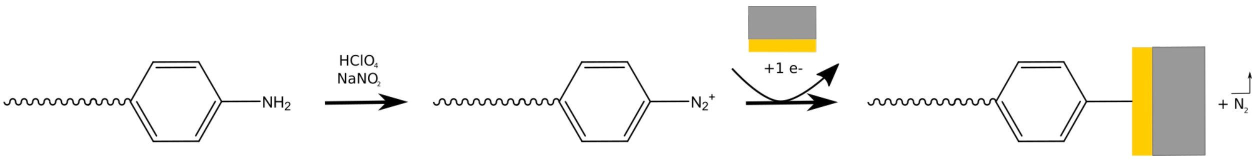

Supplementary Figure 3: **Principle of grafting of PMeOx by electroreduction of diazonium salts.** Grafting steps: the diazonium salt is generated in situ from the aminophenyl group on the polymer, in presence of an acid ($HClO_4$) and of a nitrite compound ($NaNO_2$); the reduction of the diazonium salt establishes a bond with gold.

(1mM in deionized water), was flowed through the nanoporous membrane (800 Pa for 200 nm pores) for 20 min before performing the experiments. Depending on the hydrophobicity of the surface to be passivated, the arrangement of the Pluronic layer is different and can range from pancake-like (hydrophilic surface) to brush-like configurations (hydrophobic surface)[21]
.

Zero-Mode Waveguide Detection

Experimental Setup

The experimental setup is based on a near field optical setup that combined fluorescence microscopy and the Zero-Mode Waveguide effect. The experimental setup consisted of two chambers separated by a nanoporous membrane. The cis chamber was connected to a pressure controller (microcontroller MFCS, Fluigent) and it contained the viral particles that were fluorescently labelled. The application of a pressure difference between the two chambers, allowed the transport of the viral particles in the trans chamber. The membrane was illuminated on the gold side by a laser (wavelength of 473 nm) and the events were observed at the exit of the pores. After, they became either unfocused or bleached. The inverted microscope (Axiovert 200) was connected to an electron multiplying charge coupled device camera (EMCCD camera, Andor, iXon 897). The setup also comprised a water objective (ZEISS C-Apochromat,63x magnification, 1.2 numerical aperture). Signal to noise enhancement in the vicinity of the nanopore end was superior to 2 for all types of particles that

were studied (see[5]). Our measurements were performed in real time and time resolution was determined by the acquisition frequency of the camera (30 ms for an Andor EMCCD iXon 897). The resolution of the image was 512 x 512 pixels in 32 bits, and the observed area corresponds to $125 \times 125 \mu m^2$.

More details on Zero-Mode Waveguide, fluorescence enhancement, and electromagnetic field modelling are available in the Supporting Information of our previous articles[5,9,18,22] .

Image Analysis and Fitting Procedure

From 1 to 200 translocation events were observed on each movie (250 frames, 33 fps). The events were counted by eye (manual counting) to obtain the number of events on a film, noted N. Then, the translocation frequency can be deduced as follows:

$$f = \frac{N \times f_{acquistion}}{\sigma \times S \times N_{frames}} \quad (1)$$

With $f_{acquisition} = 33$ fps the acquisition frequency, σ the pore density (see Supplementary Table 1), $S = 125 \times 125$ μm^2 the observed area, and $N_{frames} = 250$, the number of frames. We recorded each point in quadruplet over different experiments (between 8 and 36 replicates), that were then averaged to obtain the results. The errors bars were determined from the 95% standard error ($\pm 2 \times \sigma/\sqrt{N}$ with σ the standard deviation and N, the number of measurements). The robustness of the fitting procedure was assessed by bootstrapping. Error propagation was also used.

Supplementary Discussion

Comparison with Electrical Nanopore Measurements

The jamming phenomenon, investigating in this article, differed significantly from what can occur in electrical nanopore measurements. The crucial difference lied in the process reversibility. In the present case, we observed no hysteresis during the measurement of translocation frequency versus pressure/concentration, whereas in electrical detection, it typically requires pore replacement due to complete blockage.[23]

Regarding concentration range, in our experiments concentration typically varied between $10^5 - 10^7$ particles/mL. The maximum concentration that we have reached is 1.7×10^8 for AAV-8 particles (see Figure 5 in the article). For concentrations in this range, we observed the same jamming behaviour. Overall, we have not explored concentrations exceeding 10^8 particles/mL and cannot rule out the possibility of irreversible binding within this range of concentration.

Nevertheless, in comparison electrical detection methods often operate at higher concentration ($10^8 - 10^{10}$ particles/mL). This limitation is mainly due to the use of a single pore in case of electrical detection versus 10^4 pores in parallel in our case with optical detection. More precisely, the nanoporous membrane that we used are track-etched membranes exhibiting very high pore densities ($1 - 3 \times 10^6$ $pores/cm2$). They are made of polycarbonate originally used for water filtration and designed to exhibit a low retention of proteins contrary to Silicium Nitride (SiN) membranes used for electrical detection. Furthermore, we also passivated the surface with Fetal Bovin Serum (FBS) in order to further limit the potential interaction between viruses and the pores.

Overall, we have highlighted the fact that the conditions used in this article were coherent with the concentration range encountered in biological contexts. Indeed, the viral concentrations encountered in biological conditions, such as patient biofluids, can vary from 10^3 to 10^7 particles/mL, depending on various factors such as the type and stage of infection.[24]

This alignment underlines the relevance and applicability of our findings to understanding biological mechanisms and strengthens the translational potential of our research.

Nanobead Translocation through Nanopores

To improve comparability between the surface state of the two nanobead types, we carried out complementary experiments with nanobeads and nanopores of different sizes. We performed experiments with both smaller ($R_{beads} = 38 \pm 11nm$) and larger ($R_{beads} = 130 \pm 35nm$) carboxyl beads than the amino beads used in the article ($R_{beads} = 85 \pm 25nm$), using the same pore diameter (400 nm). As illustrated in Supplementary Figure 4.A, a linear evolution of the translocation frequency with pressure for different concentration was observed with the carboxyl nanobeads of two sizes in large pores (400 nm). The same behaviour was observed for the small carboxyl beads ($R_{beads} = 38 \pm 11nm$) in smaller pores (200 nm) as represented in the article (Figure 2.A). As for amino beads (Figure 1.B & Supplementary Figure 4.B), the appearance of a critical pressure, similarly to the one observed for viruses, was evidenced. However, there was no saturation at high pressures, and the increase in concentration was accompanied by an increase in translocation frequency. , these additional experiments (Supplementary Figure 4.A) showed that the size of the pore and of the particle didn't affect the linear regime observed in the case of carboxyl beads (as long as the bead radius remains smaller than the pore radius). The difference in behaviour observed during the transport of carboxyl (Supplementary Figure 4.A) versus amino (Supplementary Figure 4.B) beads was therefore not due to a difference in size but rather to electrostatic and hydrophobic interactions.

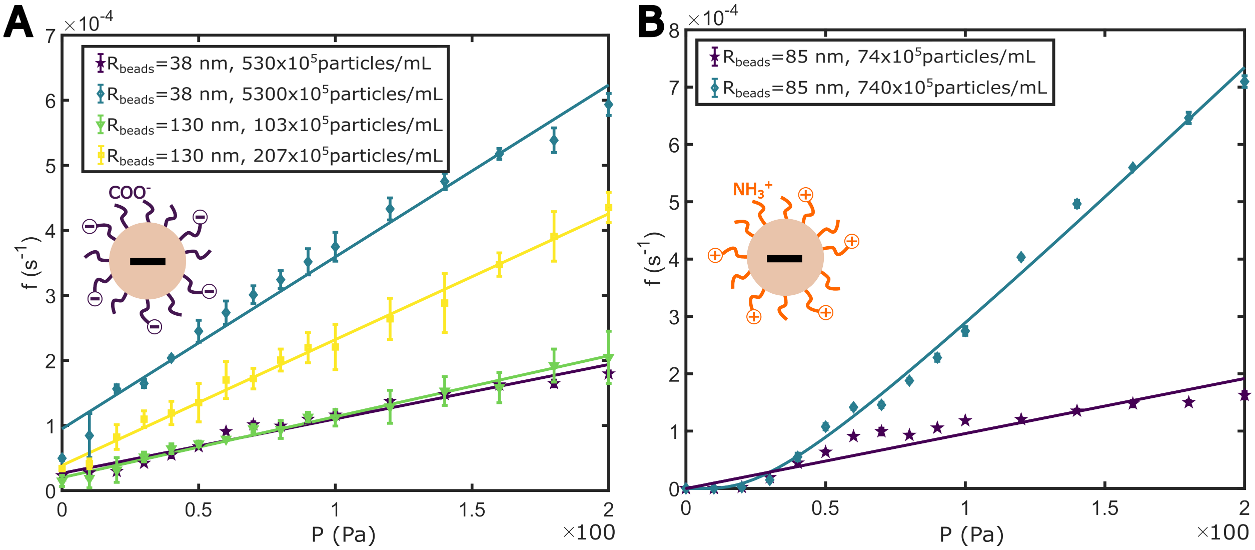

Supplementary Figure 4: **A. Translocation frequency as a function of pressure for polystyrene nanobeads functionalized with carboxyl groups of two sizes at different concentrations.** Linear regimes were observed (continuous color lines) contrary to viral particles. The two carboxyl beads have respective radius of: $R_{beads} = 38 \pm 11nm$, measured by DLS and $R_{beads} = 130 \pm 35nm$, provided by the supplier (Spherotech Inc). **B. Translocation frequency as a function of pressure for polystyrene nanobeads functionalized with amino groups at different concentrations.** Presence of a critical pressure (below which no nanobeads were observed) followed by a linear regime. Continuous color lines were fitted by $k^{-1}\frac{P_c}{P}exp(\frac{P_c}{P})$ (see τ_2 in equation (4) in the article). $R_{beads} = 85 \pm 25nm$, provided by the supplier. **For A. and B.** Pore diameter 400 nm. Experimental errors were the standard error of the mean, and for each experimental series there were N=24 technical replicates.

Comparison between Entry and Exit Experiments

We performed complementary experiments to validate the comparability of "exit" and "entry" experiments.

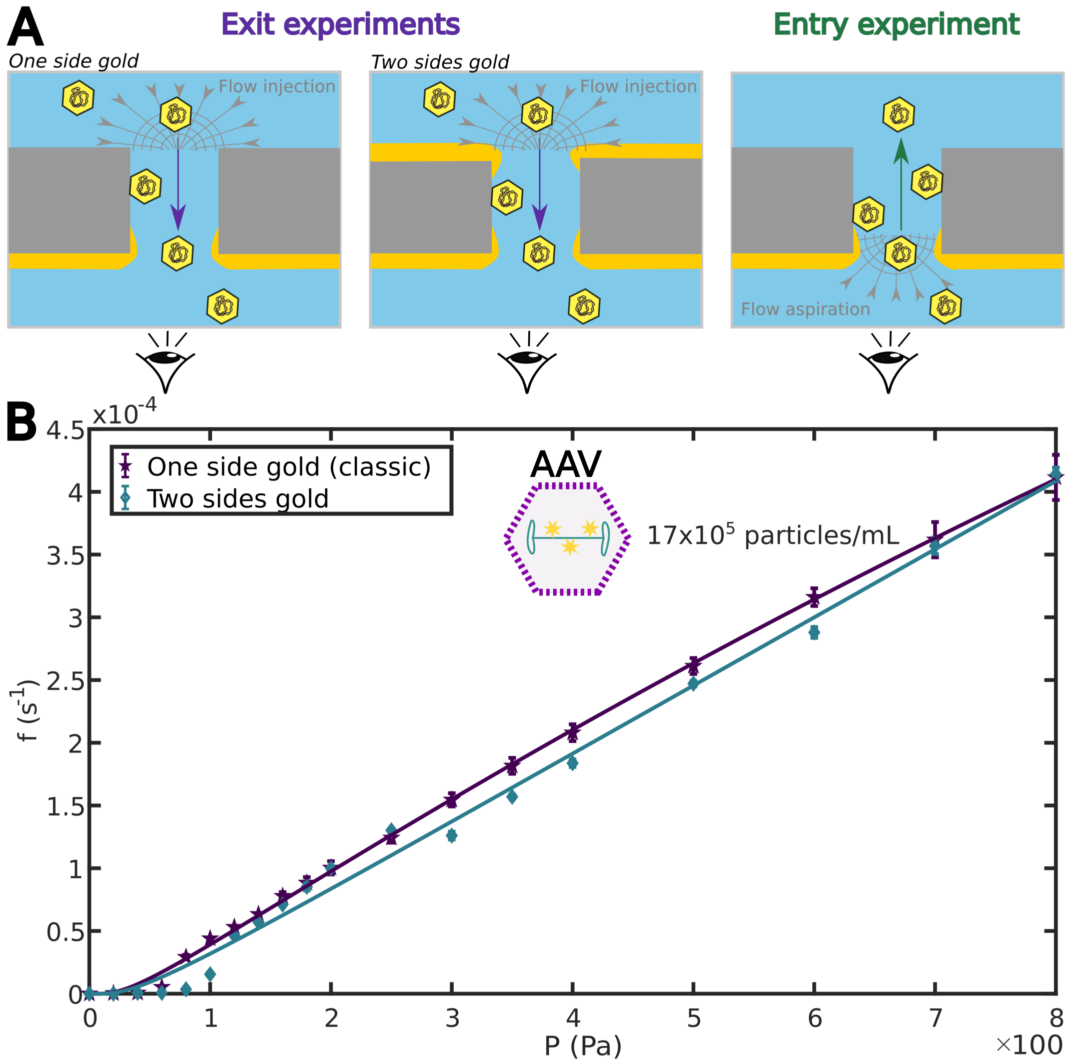

Supplementary Figure 5: **A. Different experimental configurations.** (Left) Exit experiment (with one side gold); (Middle) Exit experiment (with two sides gold); (Right) Entry experiment (with one side gold). The configuration used conventionally is the one on the left.**B.Comparison between a classic membrane and a recto/verso (coated with gold) membrane.** Translocation frequency of AAV-8 particles (17.10^5 particles/mL) as a function of applied pressure. Classic membrane: only exit side is coated with gold (50 nm-thick). Recto/verso membrane: both entry and exit side were coated with gold (50 nm-thick). Experiments were performed by looking at the exit side. Polycarbonate VS gold at entry had no significant impact on virus transport. Pore diameter 200 nm. Experimental errors were the standard error of the mean, and for each experimental series there were N=16 technical replicates.

”Exit experiments” refer to experiments conducted by observing the exit of virus translocation, where gold is positioned on the exit side and polycarbonate at the entry side. Conversely, ”entry experiments” refer to those performed by observing the entry of viruses into the nanopores, with gold located on the entry side and polycarbonate at the exit side. Indeed, gold is always needed on the observation side to take advantage of the Zero Mode Waveguide effect. This distinction is illustrated in Supplementary Figure 5.A.

Therefore, we had to evaluate the impact for virus transport of having either gold (for ”entry experiments”) or polycarbonate (for ”exit experiments”) on the entry side. The membranes were usually covered by a nanometric layer of gold on a single side of the membrane. To verify that virus transport was not affected by the fact that there was either gold or polycarbonate at entry, we evaporated gold nanometric layers (50 nm in thickness) on both sides of a nanoporous membrane. By looking at exit, we compared the translocation frequency of AAV-8 particles as a function of pressure, for polycarbonate or gold at entry, as can be seen on Supplementary Figure 5. The curves obtained were very similar, no changes have been observed. Consequently, we concluded that the choice of polycarbonate or gold at entry did not significantly affect virus transport.

Furthermore, we also focused on observing the exit/entry of viral particles from/in the pores. At present, we lack a method capable of effectively probing the internal structure of a jammed pore. Nevertheless, despite examining both entry and exit events, we did not detect the presence of aggregated particles or clogs at pore exit or entrance, as exhibited in Supplementary Figure 6. These snapshots provide valuable visual insights into the behaviour of viral particles within our exit versus entry experimental setup. The absence of visible clogs leaded us to assume that any potential blockages likely formed within the pores themselves.

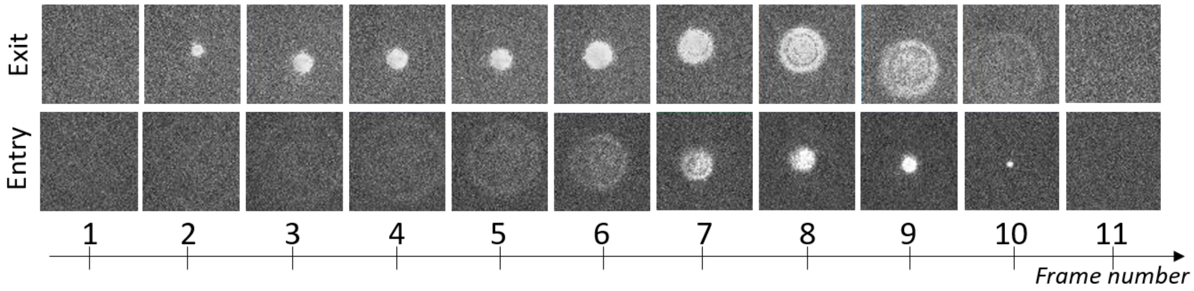

Supplementary Figure 6: **Images corresponding to the translocation of HIV (VLP) labelled using Gag-GFP.** (up) Exit experiment. (down) Entry experiment. Each line (up or down) correspond to a single event. Pores 400 nm grafted with poly(2-methyl-2-oxazoline)s. Images in chronological order, the first frame was set to 1. Image size: 15 µm. Frame rate: 33 fps.

Transient State of Clog Formation

We used a high pressure (10^4 Pa) to remove the viral clog that had formed in the central channel of the nanopore, as represented in Figure 3B in the article. At $t = 0$, the central channel was empty and the formation of the clog was followed during 5 min. The virus translocation frequency decreased exponentially, which attested to the formation of a clog and allowed us to extract a characteristic time for this phenomenon. We performed a complementary experiment (see Supplementary Figure 7) to evidenced that this phenomenon did not depend on the steady state pressure, by using 1600 Pa as steady pressure instead of 800 Pa. No significant difference was observed between the two pressures.

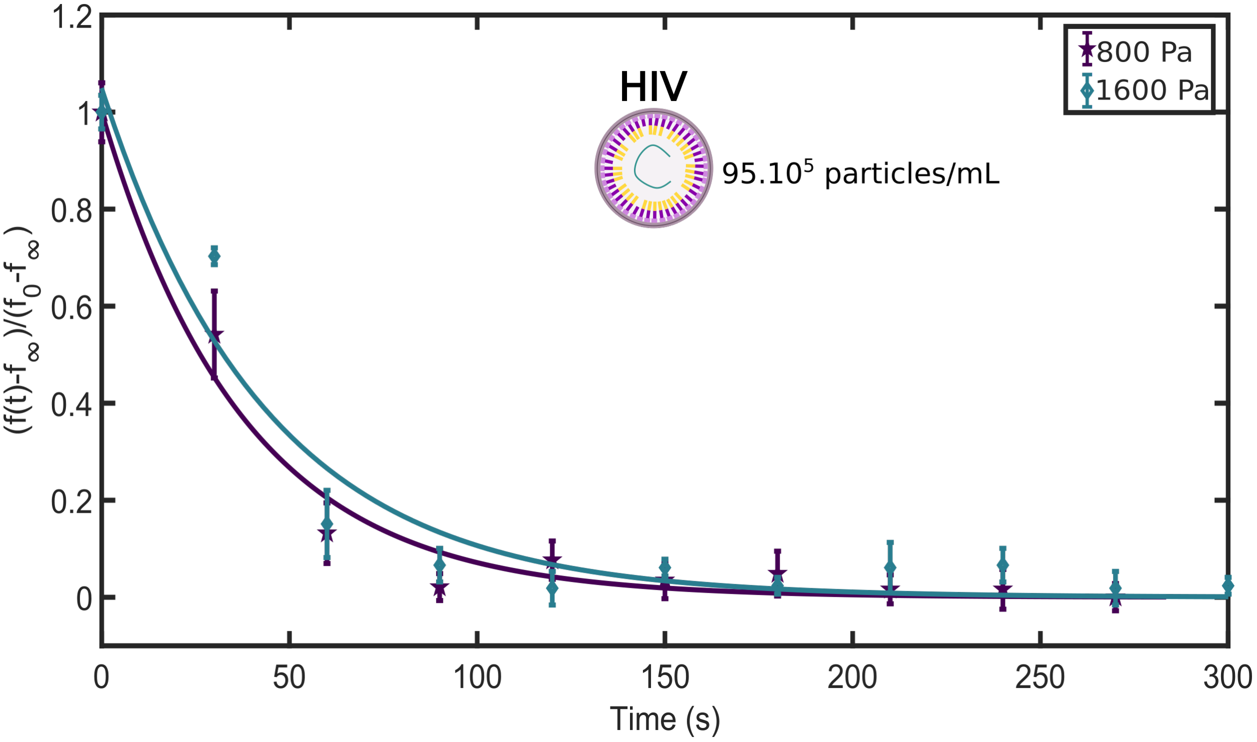

Supplementary Figure 7: **Dynamic of virus clog for different pressures of steady regime.** ($\frac{f(t)-f_\infty}{f_0-f_\infty}$) as a function of time after plug removal, for HIV particles (95.10^5 particles/mL). By using high pressure (10^4 Pa), virus plug inside the pore was removed at t =0s and the dynamic of formation of plug was followed for 300 s at a steady pressure. Two steady pressures were used (800 and 1600 Pa). Continuous color lines were fits by a decreasing exponential and we found respectively τ_{clog} equal to 38 s and 43 s for 800 Pa and 1600 Pa. Both results and experimental points were very similar. Pore diameter 200 nm. Experimental errors were the standard error of the mean, and for each experimental series there were N=16 technical replicates.

Determination of Virus Layer Thickness

In order to measure the thickness of the virus layer in the pore, we performed Zero-Mode Waveguide experiments with double stranded (ds) DNA (λ-DNA, 48 502 pb, *Invitrogen)*, whose behaviour through the nanopore is well known and described by the suction model[9,18,22] . Here, we looked at the transport of dsDNA through nanopores that were clogged by viruses. The resulting pressure-frequency curves were fitted with the suction model and the extracted critical pressures were then compared to the one originating from naked membranes (without viruses). Supplementary Figure 8 represented an example of the translocation frequencies of dsDNA as a function of pressure for different membranes (naked and with viruses), that were fitted by the suction model. Supplementary Table 3 recapitulated the obtained critical pressure values. Moreover, if we assumed that the transport through the nanopore clogged with viruses, verified the Poiseuille law, P_{suc} can be directly related to the radius of the nanopore. More precisely, in this frame, the critical pressure P_{suc} is proportional to the inverse of the pore radius R to the power of four $P_c \sim R^{-4}$ (see in the article). Therefore, a small change in the pore radius should lead to a huge difference in the critical pressure which allows to measure the thickness of a virus layer (or of grafted polymers) inside the pore (see equation (1) in the article). This type of experiments with our system has already been carried out to measure the thickness of poly(N-isopropylacrylamide)[20] or of poly(2-alkyl-2-oxazoline)s[18] grafted onto gold nanoporous membrane. Here, the compact layer hypothesis is necessary to extract the virus layer thickness from the critical pressure (Pc) originating from the suction model (Section II.B of the article). This is a simplifying hypothesis to get information on the crowding of the pore. However, a more complex architecture of the virus aggregate may be involved.

The resulting thickness of virus clog for the curves depicted on Supplementary Figure 8 are shown in Supplementary Table 3.

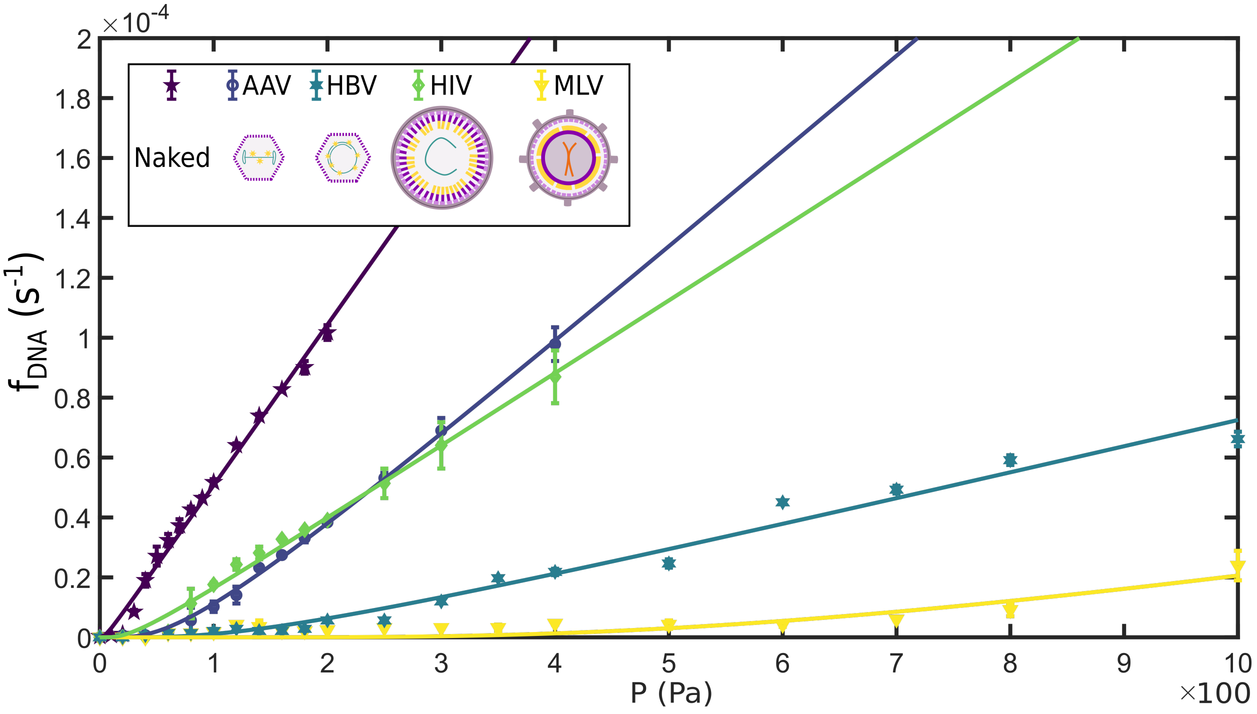

Supplementary Figure 8: **A. Transport of dsDNA through membranes without or with a virus clog.** Translocation frequency of dsDNA as a function of pressure for different conditions. Membranes were either naked or used after transport of viruses for 20 min at 800 Pa: AAV-8 (340.10^5 particles/mL), HBV (510.10^5 particles/mL), HIV (190.10^5 particles/mL) and MLV (520.10^5 particles/mL). Continuous color lines were fits by the suction model: $f_{DNA} = f_{suc}\frac{P}{P_{suc}}e^{-\frac{P_{suc}}{P}}$. Pore diameter 200 nm. Experimental errors were the standard error of the mean, and for each experimental series there were N=20 technical replicates.

Supplementary Table 3: **Calculation of the virus layer.** Experimental data in this table corresponded to the curves depicted on Supplementary Figure 8. Pore diameters 200 nm. Experimental errors for P_{suc} were calculated using jack knifing method and errors for R_e were obtained using error propagation.

Type	$P_{suc}(Pa)$	R_e (nm)
Naked	15±2	/
AAV-8	240±90	40±1
HBV	91±40	40±4
HIV	138 ±10	47±1
MLV	1200±100	73±2

Variation of NaCl Concentration

Complementary experiments on HIV (VLP) particles were carried out with concentrations of NaCl of 150, 300 and 600 mM. As depicted in Supplementary Figure 9, our findings revealed that the addition of NaCl to HIV solutions led to an increase in the translocation frequency higher and a less pronounced saturation. The behaviour appeared independent of NaCl concentration, as the obtained curved were similar for 150, 300 and 600 mM. In this case, the transport of viruses was facilitated by electric charge screening that prevented electrostatic interactions of viruses with each other or with the pore.

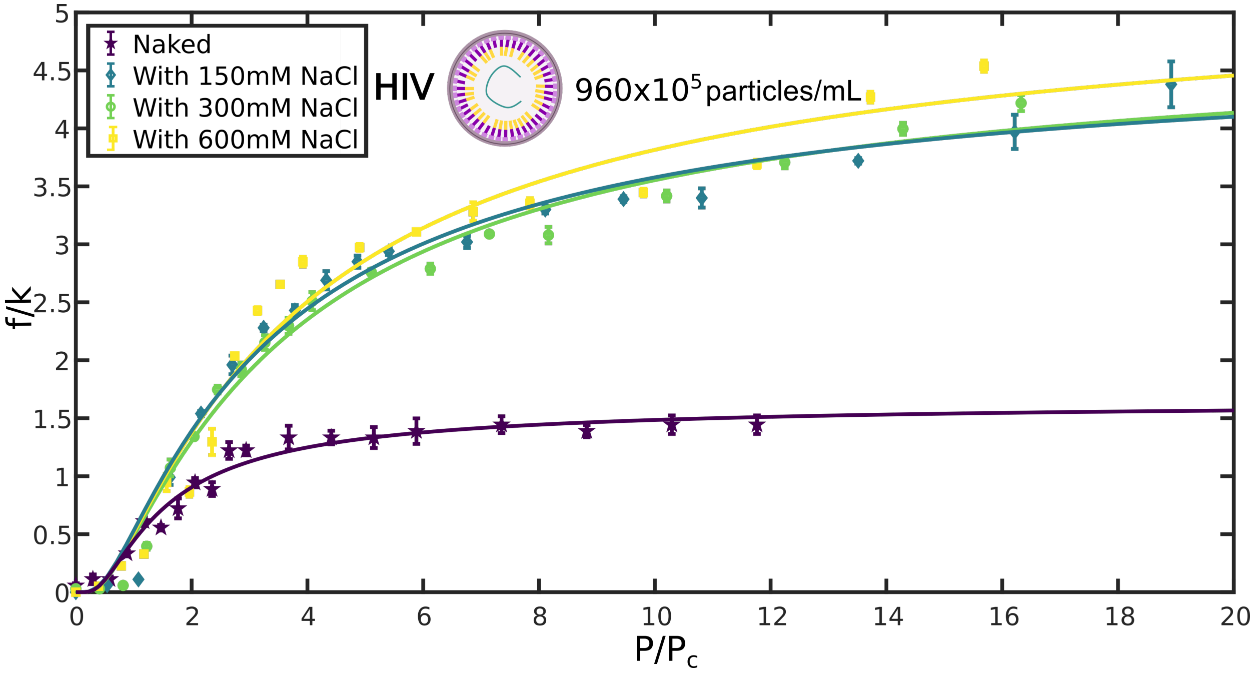

Supplementary Figure 9: **HIV translocation with different NaCl concentrations**. Evolution of normalized translocation frequency as a function of normalized pressure for HIV particles: naked or with NaCl (150 mM, 300 mM and 600 mM). The translocation frequency was higher with salt compared to the naked membrane. The saturation was also less marked in this case. Nevertheless, it did not seem to depend on NaCl concentration. Experimental errors were the standard error of the mean, and for each experimental series there were N=16 technical replicates.

Discussion of the Model

Complete Description of the Model

We proposed a simple physical model to describe the phenomenon of flow-driven jamming of viral particles in nanopores which reflected experimental observations. We assumed that advection to the pore was not a limiting step. This assumption has been used in other model such as the suction model for the transport of DNA molecules.[9] Here, the barrier is limited not by a confinement energy barrier but by the accessibility of the site as in Langmuir isotherm models. Hence, the translocation time was dominated by this barrier while the time to reach the pore was assumed to be negligible. The duration of a virus passage through a nanopore can therefore be decomposed into two independent timescales: τ_1 (virus entry duration) and τ_2 (virus translocation duration through the pore)

Firstly, we assumed that the entry duration was related to the interaction of viruses with the clog. We made the simple assumption that entry duration depended only on the probability for the pore to be occupied by other viruses (P_{virus}). Assuming that the equilibrium between viruses in solution and those associated with the clog is given by the Langmuir equilibrium, the entry time can be expressed as follows:

$$\tau_1(C) = (k_{off}^{clog})^{-1} P_{virus} = (k_{off}^{clog})^{-1} \frac{C}{C + K_d^{clog}}, \tag{2}$$

with k_{off}^{clog}, the characteristic off-rate at which virus detached from the clog and K_d^{clog}, the dissociation constant between the virus and the clog.

Secondly, concerning τ_2, the virus translocation duration through the pore, we took into account the potential adhesion of viruses to the central channel. We hypothesized that in the stationary regime, inside the nanopore, the concentration of viruses, $C_n(x,t)$, can be described as an interplay between adhesion to the pore surface and advection by the driving

flow. Virus conservation may then be written as followed:

$$\frac{\partial C_n(x,t)}{\partial t} = -k_{on}^{pore} C_n(x,t) - v\frac{\partial C_n(x,t)}{\partial x} = 0, \tag{3}$$

with v, the speed of the flow and k_{on}^{pore}, the characteristic on-rate at which a virus stick to the central channel of the nanopore.

Therefore, the concentration of viruses in the nanopore can be expressed as:

$$C_n(x,t) = Cexp(-\frac{k_{on}^{pore}}{v}x), \tag{4}$$

With C, the concentration of particles in the upstream chamber (at x=0).

To introduce our experimental control parameter, i.e the pressure difference between the two chambers (P), we assumed that the transport though a nanopore was dominated by advection and that the flow was predicted by the Poiseuille law (see below). In our cylindrical geometry, $v = \frac{P}{\pi R^2 R_h}$, with $R_h = \frac{8\eta L}{\pi R^4}$, where R and L were respectively the radius and the length of the pore, and η, was the water viscosity.

Therefore, the translocation duration trough the nanopore (τ_2) can be expressed as follows:

$$\tau_2(C,P) = \frac{1}{\pi R^2 v C_n(L,t)} = \frac{1}{\pi R^2 v C} exp(\frac{k_{on}^{pore}}{v}L) = k^{-1}\frac{P_c}{P}exp(\frac{P_c}{P}), \tag{5}$$

introducing a prefactor $k = \pi k_{on}^{pore} CLR^2$ and a critical pressure $P_c = \frac{8\eta k_{on}^{pore} L^2}{R^2}$.

Finally, the translocation frequency of a virus through a nanopore can be written as:

$$f(C,P) = \frac{1}{\underbrace{\tau_1(C)}_{\text{entry}} + \underbrace{\tau_2(C,P)}_{\text{exit}}} = \frac{k}{\frac{k}{k_{off}^{clog}}\frac{C}{C+K_d^{clog}} + \frac{P_c}{P}e^{\frac{P_c}{P}}} \tag{6}$$

Evaluation of Competition between Advection, Diffusion and Binding

If we take into considerations all the pressures, pore size and both viral particle and bead sizes, we found a Peclet greater than one :$P_e = \frac{vL}{D}$ (with v the speed of the flow through a pore, L the length of the pore, and $D = \frac{k_B T}{6\pi\eta R_{particle}}$ the diffusion coefficient of virus or nanobeads). More precisely, Peclet number varied between: $2 < P_e < 400$ and for viral particles in the saturation regime it was between $10 < P_e < 400$.

Therefore, in the simple case of a competition between advection and diffusion, advection would dominate. This is the case for negatively charged nanobeads (see Figure 2.A in the article). However, when additional interactions such as particle aggregation, binding and unbinding to the pore surface are present, this consideration should be completed by an estimate of the ratio of binding rate over advection rate. For example the competition between binding and advection can be estimated with the advection-binding number $(\frac{k_{on}^{pore} L}{v})$. In the geometry of our experiments, this number is ranging between 0.05 and 3 for the pressure range used in our experiments showing that we operate at a cross over between binding and advection. Unfortunately, the determination of all the dynamical parameters (binding, unbinding to the pore and the other viruses) is inaccessible with our experiments.

Prefactor k Determination

Prefactor k is a numerical factor in equation (4) of the article. It depends on experimental conditions such as the number of active pore on the observed surface. This number of active pore can vary from an experiment to another in particular from one membrane to another. Prefactor k may also depends on virus concentration. For these reasons, we determined k for each experimental condition with the following protocol. At low pressures, before the saturation phenomenon, the experimental measurements of the translocation frequency as a function of pressure were fitted by $f = \frac{1}{\tau_2} = k\frac{P}{P_c}e^{\frac{-P_c}{P}}$. It allowed us to extract both the critical pressure P_c and the prefactor k. An example of raw data for HIV VLP, including a fit with $\frac{1}{\tau_2}$ is given in Figure 10.

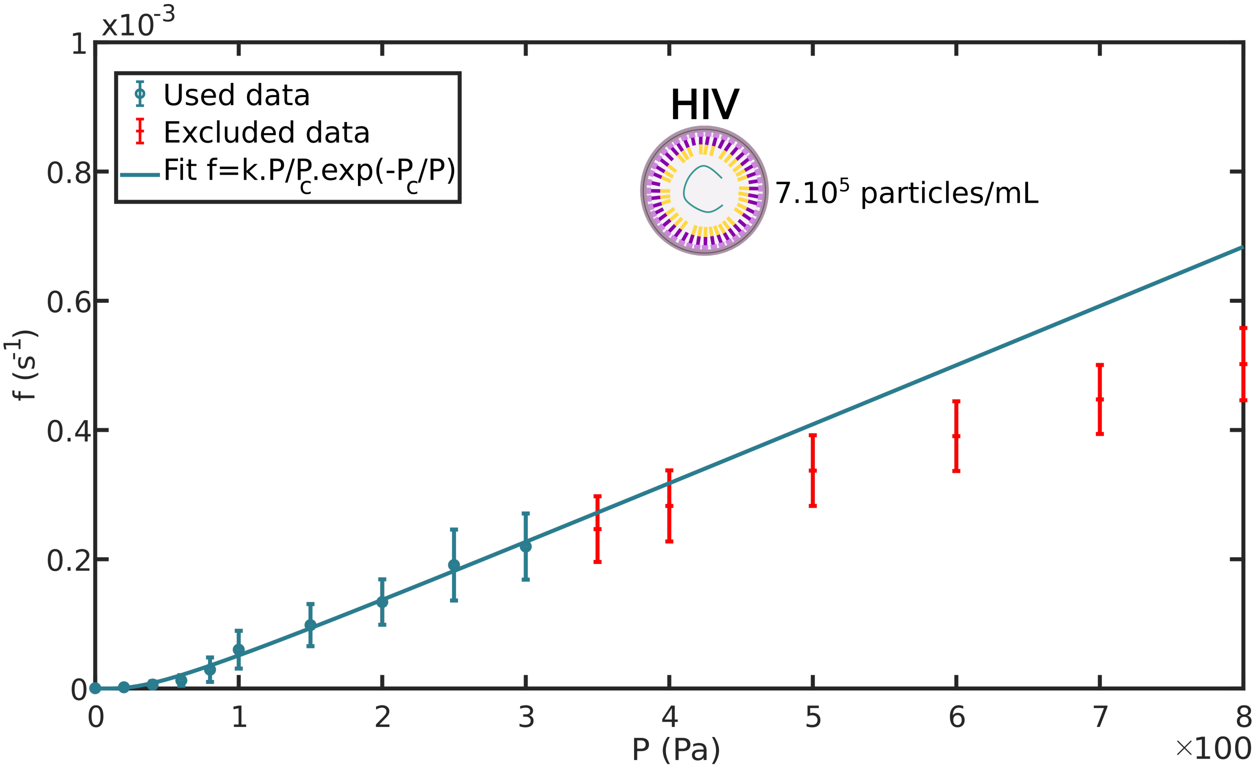

Supplementary Figure 10: **Raw data for HIV (VLP) frequency versus pressure curve.** Translocation frequency of HIV (VLP) particles (7.10^5 particles/mL) as a function of applied pressure. Used data (in blue) were fitted by $f = \frac{1}{\tau_2} = k\frac{P}{P_c}e^{\frac{-P_c}{P}}$. Excluded data (in red) were in the saturation regime and were not taken into account for the fit. Extracted parameters from the fit (P_c and k) are given in Supplementary Table 4. Experimental errors were the standard error of the mean, and for this experimental serie there were N=12 technical replicates.

Moreover, examples of numerical values for P_c and k are recapitulated in Supplementary Table 4 for HIV (VLP). It can be observed that P_c did not depend on concentration, unlike the prefactor k. Because of the uncertainty on the measurement of k we didn't use this parameter to determine k_{on}^{pore}.

Supplementary Table 4: **Parameters Pc and k for HIV (VLP) particles.** Experimental data summarized in this table corresponded to parameters P_c and k extracted from the fit with $f = \frac{1}{\tau_2} = k\frac{P}{P_c}e^{\frac{-P_c}{P}}$, as described in the method above. Pore diameter 200 nm. Experimental errors for P_c and k were calculated using jack knifing method.

Concentration (particles/mL)	7.10^5	14.10^5	35.10^5	70.10^5	350.10^5	500.10^5
P_c (Pa)	55 ± 20	60 ± 30	43 ± 20	49 ± 30	47 ± 30	45 ± 10
k ($\times 10^{-5} s^{-1}$)	5 ± 3	1 ± 0.5	2 ± 1	3 ± 2	15 ± 6	130 ± 30

Master Curves

In order to highlight the fact that the total translocation time can be decomposed into two times (entry, τ_1 and translocation duration, τ_2), we have drawn two curves:

- Supplementary Figure 11.A: the first graph corresponds to $\tau_1 \times k$ ($\tau_1 \times k = (k_{off}^{clog})^{-1}\frac{C}{C+K_d^{clog}} \times k$) as a function of concentration for different pressures. τ_1 was obtained by considering the total translocation time τ, to which we subtracted the constant $\frac{P_c}{P}exp(\frac{P_c}{P})$, which was fixed for each pressure curve. Experimental data for 9 different pressures were represented and the black continuous line corresponded to the jamming model.

- Supplementary Figure 11.B: the second graph corresponds to $\tau_2 \times k$ ($\tau_2 \times k = \frac{P_c}{P}exp(\frac{P_c}{P})$) as a function of pressure for different concentrations. τ_2 was obtained by considering the total translocation time τ, to which we subtracted the constant $(k_{off}^{clog})^{-1}\frac{C}{C+K_d^{clog}}$, which was fixed for each concentration curve. Experimental data for 5 different concentrations were represented and the black continuous line corresponded to the jamming model.

In both cases, the data fell on each other as expected, and the model fitted rather well the data. Multiplications by the prefactor k (depending on the concentration and on the number

of active pore, see above) allowed us to have two decorrelated terms, the first depending only on the concentration and the second on the pressure.

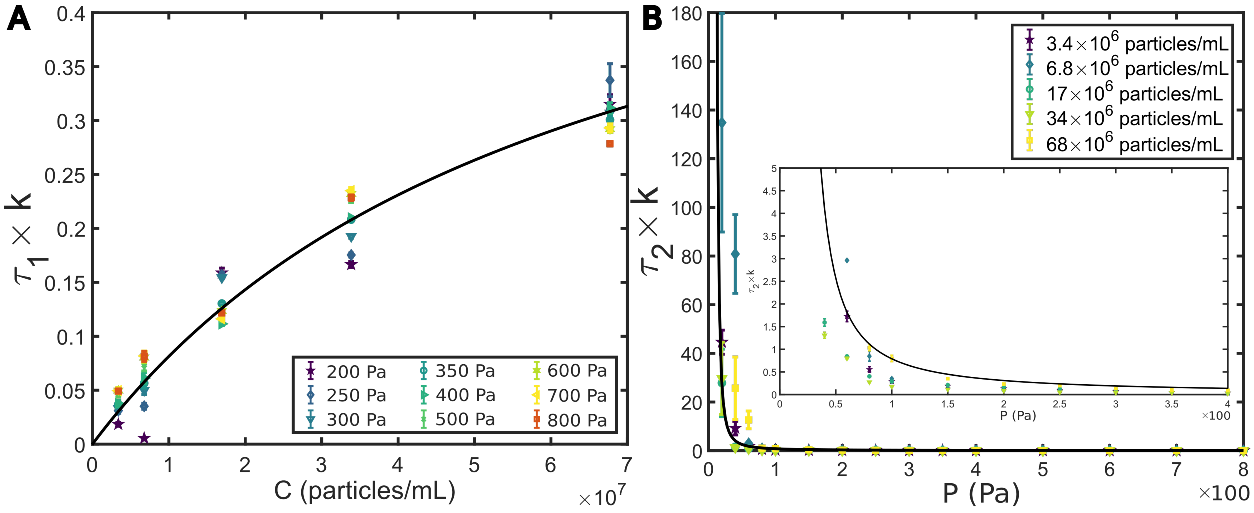

Supplementary Figure 11: **Representation of the two times of the jamming model: entry and translocation duration.** A. Entry term: $\tau_1 \times k$ as function of concentration for different pressures. Continuous black line represented the jamming model. B. Translocation duration term: $\tau_2 \times k$ as function of pressure for different concentrations. Inset: zoom of the graph. Continuous black lines represented the jamming model. Experimental data were obtained for AAV-8 particles through pores of 200 nm diameter. Experimental errors were the standard error of the mean, and for each experimental series there were N=16 technical replicates.

Other Experiment Types

Zeta Potential

We have carried out additional experiments using a zetameter (Zetasizer-Nano ZS, Malvern Panalytical) from the Laboratoire de Chimie (ENS de Lyon) to measure the zeta potential of the different nanobead types of the same size. As depicted in Supplementary Table 5, our findings revealed that the carboxyl beads (R_{beads} = 80 nm) exhibited a negative zeta potential of -63.8 mV, while the amino beads (R_{beads} = 85) showed a zeta potential of -54.8 mV.

Supplementary Table 5: **Measurement of zeta potential of the different nanobead type.** Experiments were performed in Tris-EDTA buffer. Concentrations were respectively of 4.4×10^{10} particles/mL for carboxyl nanobeads and of 3.7×10^{10} particles/mL for amino nanobeads. Values were averaged over a minimum of 2 measurements.

Nanobead type	Zeta Potential (mV)
Carboxyl nanobeads ($R_{beads} = 80 \pm 20$ nm)	-63.8±9.6
Amino nanobeads ($R_{beads} = 85 \pm 25$ nm)	-54.8±2.4

The negative values for the zeta potential of both carboxyl and amino nanobeads may be explained by the low density of the amino groups grafted on the particle (10^4 amino groups per particles) which can compensate for the negative charges (carboxyl group mainly) initially present on the particle before functionalization. This measurement emphasizes that the pressure threshold observed for amino nanobeads is due to short range surface interactions.

Regarding the nanoporous membrane, measuring its zeta potential using commercial techniques is not feasible. However, scientific literature sources have provided some relevant data. Notably, R. Paoli et al. measured the zeta potential for a polycarbonate track etched membrane, corresponding to a value of -12 mV.[25] They also provided interesting information about surface charge density of such membranes (about -11 mC/m^2). The membrane used

are very similar to the ones we are using therefore making a suitable comparison. Additionally, complementary studies have reported zeta potential values for track-etched membranes made from different materials, such as poly(ethylene terephthalate), which showed a value of approximately -40 mV.[26]

Bio-Layer Interferometry

Bio-Layer Interferometry is a technique intended for measuring biomolecular interactions. It is based on the analysis of the interference pattern of white light reflected from two surfaces: an internal reference layer (biosensor tip) and a layer corresponding to the potentially immobilized particles on the biosensor tip. Changes in the number of particles bounded to the biosensor tip generate a shift in the interference pattern, that can be measured in real-time. It provides an opportunity to investigate association and dissociation steps and the response curve provides access to the associated kinetic parameters.

Here, we performed experiments at the Protein Science Facility at the Institut de Biologie et Chimie des Protéines (IBCP, Lyon, France) and we used the BLItz apparatus from ForteBio combined with the APS biosensor kit provided by Sartorius. The biosensor tip corresponded to an hydrophobic surface (functionnalized with aminopropyl silane) that can mimic the hydrophobic surface of the nanopore. After hydrating an APS biosensor in 200 μL PBS buffer during 10 minutes, we performed a kinetic experiments on AAV-8 and proceeded as follows:

- Baseline: biosensor tip in 250 μL PBS buffer (shaker at 1000 rpm)
- Association: biosensor tip in 4 μL in AAV-8 sample (concentration 1.4×10^{12} particles/mL, shaker at 1000 rpm)
- Dissociation: biosensor tip in 250 μL PBS buffer (shaker at 1000 rpm)

A similar protocol was followed to make a reference sample, where the AAV-8 sample was replaced by PBS buffer. The resulting curves were depicted in Supplementary Figure 12,

where the three steps (baseline, association and dissociation) were evidenced.

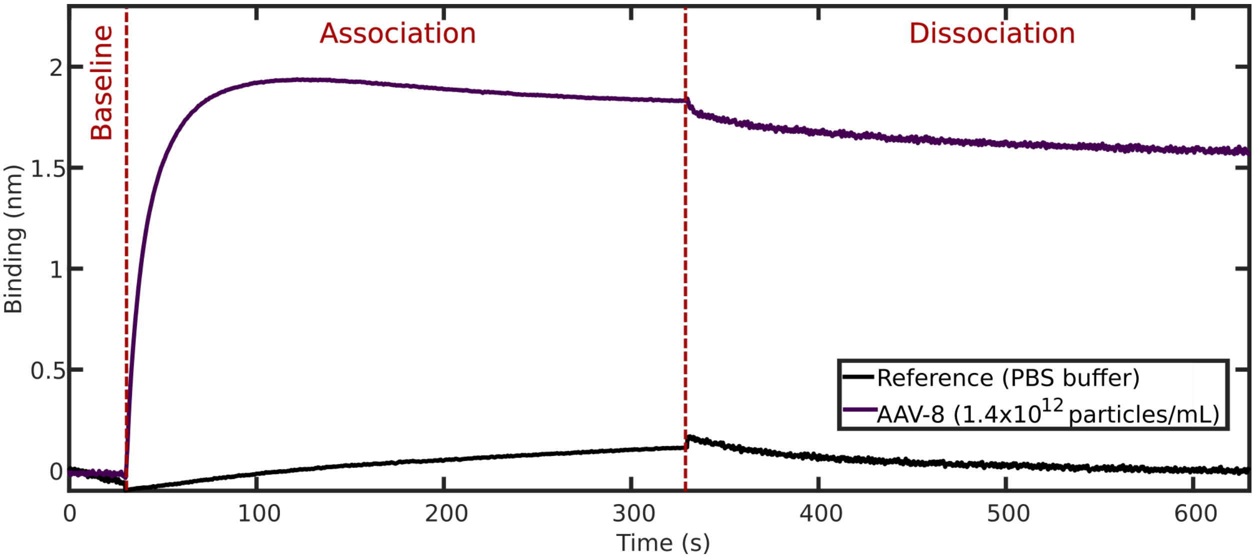

Supplementary Figure 12: **BLI curves for the interaction between APS biosensor and AAV-8.** Points corresponded to experimental data: black points corresponded to the reference sample and violet points corresponded to the AAV-8 sample (1.4×10^{12} particles/mL). The three steps (baseline, association and dissociation) were evidenced.

After subtracting the reference sample from the AAV-8 curve, fits by a simple increasing exponential (association) and decreasing exponential (dissociation) were performed on the resulting curve (not shown on Figure 12). The extracted parameter are the dissociation rate constant ($k_{off} = 1.1 \pm 0.3 \times 10^{-2}$ s^{-1}), the affinity constant ($K_d = 1.1 \pm \times 10^{-9}$ M) and the association rate constant ($k_{on} = 0.1 \pm 0.05$ s^{-1}). This association rate constant, obtained by BLI, was representative of the interaction between AAV-8 and the hydrophobic APS surface of the biosensor. This value was comparable to the characteristic on-rate (or association constant) obtained, with our method, for the interaction between AAV-8 and the hydrophobic surface of the nanoporous polycarbonate membrane (for AAV-8, $k_{on}^{pore} = 0.5 \pm 0.2$ s^{-1}).

References

(1) Nydegger, S.; Foti, M.; Derdowski, A.; Spearman, P.; Thali, M. HIV-1 egress is gated through late endosomal membranes. *Traffic* **2003**, *4*, 902–910.

(2) Chamontin, C.; Rassam, P.; Ferrer, M.; Racine, P.-J.; Neyret, A.; Lainé, S.; Milhiet, P.-E.; Mougel, M. HIV-1 nucleocapsid and ESCRT-component Tsg101 interplay prevents HIV from turning into a DNA-containing virus. *Nucleic Acids Research* **2015**, *43*, 336–347.

(3) Jouvenet, N.; Neil, S. J. D.; Zhadina, M.; Zang, T.; Kratovac, Z.; Lee, Y.; McNatt, M.; Hatziioannou, T.; Bieniasz, P. D. Broad-Spectrum Inhibition of Retroviral and Filoviral Particle Release by Tetherin. *Journal of Virology* **2009**, *83*, 1837–1844.

(4) Salvetti, A.; Grève, S.; Chadeuf, G.; Favre, D.; Cherel, Y.; Champion-Arnaud, P.; David-Ameline, J.; Moullier, P. Factors influencing recombinant adeno-associated virus production. *Human Gene Therapy* **1998**, *9*, 695–706.

(5) Chazot-Franguiadakis, L.; Eid, J.; Socol, M.; Molcrette, B.; Guégan, P.; Mougel, M.; Salvetti, A.; Montel, F. Optical Quantification by Nanopores of Viruses, Extracellular Vesicles, and Nanoparticles. *Nano Letters* **2022**, *22*, 3651–3658.

(6) Rye, H. S.; Yue, S.; Wemmer, D. E.; Quesada, M. a.; Haugland, R. P.; Mathies, R. a.; Giazer, A. N. bis-intercalating asymmetric cyanine dyes : properties and applications CO-. *Nucleic acids research* **1992**, *20*, 2803–2812.

(7) Netzel, T. L.; Nafisi, K.; Zhao, M.; Lenhard, J. R.; Johnson, I. Base-content dependence of emission enhancements, quantum yields, and lifetimes for cyanine dyes bound to double-strand DNA: Photophysical properties of monomeric and bichromophoric DNA stains. *Journal of Physical Chemistry* **1995**, *99*, 17936–17947.

(8) Fürstenberg, A.; Julliard, M. D.; Deligeorgiev, T. G.; Gadjev, N. I.; Vasilev, A. A.; Vauthey, E. Ultrafast excited-state dynamics of DNA fluorescent intercalators: New insight into the fluorescence enhancement mechanism. *Journal of the American Chemical Society* **2006**, *128*, 7661–7669.

(9) Auger, T.; Mathé, J.; Viasnoff, V.; Charron, G.; Di Meglio, J. M.; Auvray, L.; Montel, F. Zero-mode waveguide detection of flow-driven DNA translocation through nanopores. *Physical Review Letters* **2014**, *113*, 1–5.

(10) Molcrette, B. Transport directionnel dans un nanopore. Ph.D. thesis, ENS de Lyon, 2021.

(11) Reuter, M.; Dryden, D. T. The kinetics of YOYO-1 intercalation into single molecules of double-stranded DNA. *Biochemical and Biophysical Research Communications* **2010**, *403*, 225–229.

(12) Günther, K.; Mertig, M.; Seidel, R. Mechanical and structural properties of YOYO-1 complexed DNA. *Nucleic Acids Research* **2010**, *38*, 6526–6532.

(13) Kundukad, B.; Yan, J.; Doyle, P. S. Effect of YOYO-1 on the mechanical properties of DNA. *Soft Matter* **2014**, *10*, 9721–9728.

(14) Mougel, M.; Akkawi, C.; Chamontin, C.; Feuillard, J.; Pessel-Vivares, L.; Socol, M.; Laine, S. NXF1 and CRM1 nuclear export pathways orchestrate nuclear export, translation and packaging of murine leukaemia retrovirus unspliced RNA. *RNA Biology* **2020**, *17*, 528–538.

(15) Voelkel, C.; Galla, M.; Maetzig, T.; Warlich, E.; Kuehle, J.; Zychlinski, D.; Bode, J.; Cantz, T.; Schambach, A.; Baum, C. Protein transduction from retroviral Gag precursors. *Proceedings of the National Academy of Sciences* **2010**, *107*, 7805–7810.

(16) Ormo, M.; Cubitt, A.; Kallio, K.; Gross, L.; Tsien, R.; Remington, S. J. Crystal structure of the Aequorea victoria green fluorescent protein. *Science* **1996**, *273*, 1392–1395.

(17) Plet, L.; Delecourt, G.; Hanafi, M.; Pantoustier, N.; Pembouong, G.; Midoux, P.; Bennevault, V.; Guégan, P. Controlled star poly(2-oxazoline)s: Synthesis, characterization. *European Polymer Journal* **2020**, *122*, 109323.

(18) Kolbeck, P. J.; Benaoudia, D.; Chazot-Franguiadakis, L.; Delecourt, G.; Mathé, J.; Li, S.; Bonnet, R.; Martin, P.; Lipfert, J.; Salvetti, A.; Boukhet, M.; Bennevault, V.; Lacroix, J.-C.; Guégan, P.; Montel, F. Thermally Switchable Nanogate Based on Polymer Phase Transition. *Nano Letters* **2023**, *23*, 4862–4869.

(19) Benaoudia, D.; Nguyen, V.-Q.; Bennevault, V.; Martin, P.; Montel, F.; Guégan, P.; Lacroix, J.-C. Direct Electrografting of Poly(2-alkyl-2-oxazoline)s on Gold, ITO, and Gold Nanoparticles for Biopassivation. *ACS Applied Nano Materials* **2023**, *6*, 16267–16275.

(20) Yong, H.; Molcrette, B.; Sperling, M.; Montel, F.; Sommer, J. U. Regulating the Translocation of DNA through Poly(N-isopropylacrylamide)-Decorated Switchable Nanopores by Cononsolvency Effect. *Macromolecules* **2021**, *54*, 4432–4442.

(21) Nejadnik, M. R.; Olsson, A. L.; Sharma, P. K.; Van Der Mei, H. C.; Norde, W.; Busscher, H. J. Adsorption of pluronic F-127 on surfaces with different hydrophobicities probed by quartz crystal microbalance with dissipation. *Langmuir* **2009**, *25*, 6245–6249.

(22) Molcrette, B.; Chazot-Franguiadakis, L.; Liénard, F.; Balassy, Z.; Freton, C.; Grangeasse, C.; Montel, F. Experimental study of a nanoscale translocation ratchet. *Proceedings of the National Academy of Sciences* **2022**, *119*, 1–10.

(23) Arima, A.; Tsutsui, M.; Harlisa, I. H.; Yoshida, T.; Tanaka, M.; Yokota, K.; Tonomura, W.; Taniguchi, M.; Okochi, M.; Washio, T.; Kawai, T. Selective detections of single-viruses using solid-state nanopores. *Scientific Reports* **2018**, *8*, 1–7.

(24) Wölfel, R. et al. Virological assessment of hospitalized patients with COVID-2019. *Nature* **2020**, *581*, 465–469.

(25) Paoli, R.; Bulwan, M.; Castaño, O.; Engel, E.; Rodriguez-Cabello, J. C.; Homs-Corbera, A.; Samitier, J. Layer-by-layer modification effects on a nanopore's inner surface of polycarbonate track-etched membranes. *RSC Advances* **2020**, *10*, 35930–35940.

(26) Déjardin, P.; Vasina, E. N.; Berezkin, V. V.; Sobolev, V. D.; Volkov, V. I. Streaming potential in cylindrical pores of poly(ethylene terephthalate) track-etched membranes: variation of apparent zeta potential with pore radius. *Langmuir : the ACS journal of surfaces and colloids* **2005**, *21*, 4680–4685.
